# Uncovering adaptation with a new *Arabidopsis thaliana* multiparent intercross population

**DOI:** 10.1093/genetics/iyaf227

**Published:** 2026-01-13

**Authors:** Célia Neto, Tom P J M Theeuwen, Pádraic J Flood, Paula Unger Avila, Mehmet Göktay, Mark G M Aarts, Angela M Hancock

**Affiliations:** Molecular Basis of Adaptation Research Group, Max Planck Institute for Plant Breeding Research, Cologne 50829, Germany; Department of Plant and Environmental Sciences, University of Copenhagen, Taastrup, Copenhagen 2630, Denmark; Laboratory of Genetics, Wageningen University, Wageningen 6708PB, The Netherlands; Jan IngenHousz Institute, Wageningen University, Wageningen 6708PB, The Netherlands; Molecular Basis of Adaptation Research Group, Max Planck Institute for Plant Breeding Research, Cologne 50829, Germany; Aardaia, Wageningen 6708 WH, The Netherlands; Molecular Basis of Adaptation Research Group, Max Planck Institute for Plant Breeding Research, Cologne 50829, Germany; Molecular Basis of Adaptation Research Group, Max Planck Institute for Plant Breeding Research, Cologne 50829, Germany; Laboratory of Genetics, Wageningen University, Wageningen 6708PB, The Netherlands; Molecular Basis of Adaptation Research Group, Max Planck Institute for Plant Breeding Research, Cologne 50829, Germany; Department of Botany and Plant Pathology, Purdue University, West Lafayette, IN 47907, United States

**Keywords:** doubled haploid, multiparent intercross mapping population, population structure, Cape Verde, MPP, multiparental populations, Multiparent Advanced Generation Inter-Cross (MAGIC)

## Abstract

Understanding the molecular basis of adaptation and the genetic architecture of complex traits are longstanding goals in biology. One problem impeding this understanding is the complexity of continental populations, with their complicated demographic histories, gene flow and secondary contact. In contrast, island populations represent simpler systems where uncovering the genetic basis of complex traits and tracing how traits built up is much more tractable. In *Arabidopsis thaliana*, the Cape Verde Islands populations represent a case of long-range colonization and adaptation to a divergent selective regime. Here, we describe the development and testing of a new multiparent intercross doubled haploid population of *A. thaliana* from the Cape Verde Islands. This population balances the representation of natural diversity and overcomes the shortcomings of existing resources, such as biparental recombinant inbred lines and genome-wide association populations. Specifically, it captures variation that segregates within the archipelago but is fixed on individual islands. We mapped the genetic basis of flowering time, rosette size, and photosystem II efficiency (ΦPSII) in this inter-island intercross population, representing traits that we hypothesized may be evolving under strong selection during the colonization of the archipelago. We identified functional loci underlying these traits, including *FRI K232X* and *FLC R3X* for flowering time, and *IRT1 G130X* for ΦPSII and rosette size. Our multiparent intercross population complements existing mapping resources and provides a robust framework for investigating the genetic basis of complex traits in *A. thaliana*. This work emphasizes the value of island systems and complementary approaches for advancing our understanding of genetic adaptation.

## Introduction

Understanding the genetic architectures of traits is a fundamental goal in biology. Specific research objectivees include identifying the genetic determinants of ecologically and agronomically important traits and better understanding adaptation in the wild. To identify the genetic basis of phenotypic variation, a range of mapping approaches can be applied (C. Zhu et al. 2008; Bergelson and Roux 2010; Brachi et al. 2011; Korte and Farlow 2013; Tibbs Cortes et al. 2021; Bomblies and Peichel 2022).

To fully understand trait evolution, it is essential to integrate insights with the underlying evolutionary processes. However, reconstructing these processes is often challenging due to confounding factors inherent to natural populations, such as admixture, gene flow, and secondary contact. In this context, island systems can provide a powerful framework for studying adaptation and evolution in nature. Since Darwin and Wallace—and the beginning of the theory of evolution—island systems have been advancing our understanding of the evolutionary process. These natural experiments can be likened to evolutionary test tubes, where isolation and divergent ecological regimes create novel selective pressures for colonizing populations and species. Studying these allows researchers to understand how diversity builds up during adaptive radiations. Moreover, islands on an archipelago often function as replicates in which general evolutionary patterns can be distinguished from random drift (Wallace 1855; Darwin 1859; Losos et al. 1997; Losos and Ricklefs 2009; Grant and Grant 2014; Lamichhaney et al. 2015; Enbody et al. 2022).

The Cape Verde *Arabidopsis* system offers a particularly promising model for dissecting adaptive histories. This system combines the ecological and geographical isolation of an island population with the extensive genetic and genomic resources available for a well-characterized model organism. The Cape Verde archipelago (CVI), located between 14.80° and 17.20° North and approximately 570 km off the coast of Senegal, consists of 10 volcanic islands and is characterized by a dry tropical climate. Clarifying the evolutionary processes that led to adaptation to the challenging CVI environment could inform about how plant species may deal with future environments under climate change, including which evolutionarily and economically relevant variants may be useful to deal with such pressures, or even which evolutionary strategies enabled escape from extinction.

For over 4 decades, the only representative of *Arabidopsis thaliana* from this region was the accession Cvi-0, which has been included in several studies mainly due to its distant geographic origin and phenotypic divergence compared with more commonly studied accessions, like Col-0 or Ler-0. Over the years, Cvi-0 has been included in natural variation panels and recombinant mapping populations as a parental line. These populations, including biparental RILs (eg Cvi-0×Ler-0, Cvi-0×Col-0) and NILs (Cvi-0×Ler-0) and the multiparent AMPRILS, have been extremely valuable for discovering the underpinning of several traits (Alonso-Blanco, El-Assal, et al. 1998; Alonso-Blanco, Peeters, et al. 1998; Alonso-Blanco et al. 1999, 2005; Edwards et al. 2005; Laserna et al. 2008; O’Neill et al. 2008; Simon et al. 2008; Tessadori et al. 2009; Huang et al. 2011; Salomé et al. 2011; Kadirjan-Kalbach et al. 2019; Kim et al. 2020).

Recently, we expanded this system by sampling and sequencing natural populations of *A. thaliana* from 2 Cape Verdean islands—Fogo and Santo Antão—and reconstructed the population history of *Arabidopsis* in the archipelago. Results of this analysis indicate that Cape Verde was colonized approximately 5 to 7 kya from a North African source population, with the closest modern relatives found in Morocco. Colonization involved a small founding population (estimated at fewer than 50 individuals), resulting in a severe genetic bottleneck, with 99.9% of variation in CVI private to the archipelago, such that divergence is higher between CVI and mainland populations than between species pairs in the *Arabidopsis* genus (Fulgione et al. 2022). Population genetic inference indicates that Santo Antão was colonized first, followed by a secondary colonization of Fogo from Santo Antão. Since their split, the 2 island populations have remained genetically isolated, with 99.4% of genetic variation differentiating between the islands and no evidence of gene flow between them or with continental populations. Overall, this pattern implies that adaptation of these populations proceeded via *de novo* mutations that arose independently on each island and that genetic approaches to identify adaptive variants must take these factors into account (Fulgione et al. 2022; Tergemina et al. 2022; Elfarargi et al. 2023; Neto and Hancock 2023).

The colonizing population was exposed to a novel and harsh environment marked by low and unpredictable rainfall, with moisture available mainly as fog, resulting in a short growing season (Brochmann et al. 1997). Moreover, since CVI islands are of volcanic origin, the soil has a divergent mineral composition relative to the mainland (Brochmann et al. 1997; Tergemina et al. 2022). Previous results suggest that the adaptive response to the CVI environment involved decreased water use efficiency, likely enabling higher photosynthetic rates and more rapid growth, reduced flowering time and changes in nutrient uptake (Fulgione et al. 2022; Tergemina et al. 2022; Elfarargi et al. 2023).

Despite the knowledge gained by mapping several adaptive traits on the islands and reconstructing the evolutionary history of adaptive variants, significant challenges arose when using these natural populations: the absence of shared variation between islands hindered our ability to conduct GWAS as a whole on the archipelago, hiding potentially adaptive variants that segregate in the archipelago but are fixed or at high frequencies in one of the islands (Fulgione et al. 2022; Tergemina et al. 2022).

To reduce these limitations, we present here a new Cape Verdean *A. thaliana* multiparent intercross population that complements existing mapping resources and enables more comprehensive investigations of the genetic bases of complex traits in Cape Verde. While RIL and NIL populations, created between Cvi-0 and Eurasian lines, enable the study of the deep divergence between continental populations and the archipelago, and natural populations allow the study of within-island variation, variation that segregates in the archipelago but is fixed or at high frequency in one of the islands is not accessible through available resources. The new mapping population we present here minimizes confounding effects from demographic history, such as within and between islands population structure, breaks up accumulated linkage disequilibrium, and exposes epistatic interactions. With this study, we show how this intercross population can be successfully used to identify loci involved in adaptation to novel environments.

We hypothesized that adaptive responses to the reduced growing season and harsh edaphic environment in Cape Verde likely resulted in altered phenology, energy metabolism and nutrient uptake strategy in *Arabidopsis* populations that colonized the Cape Verde Islands. Therefore, we focused our attention on 3 potentially adaptive traits: flowering time, rosette size and photosynthetic efficiency, and characterized their genetic bases in this new intercross population.

## Materials and methods

### Creation of the multiparent intercross population

**Fig. 1.**
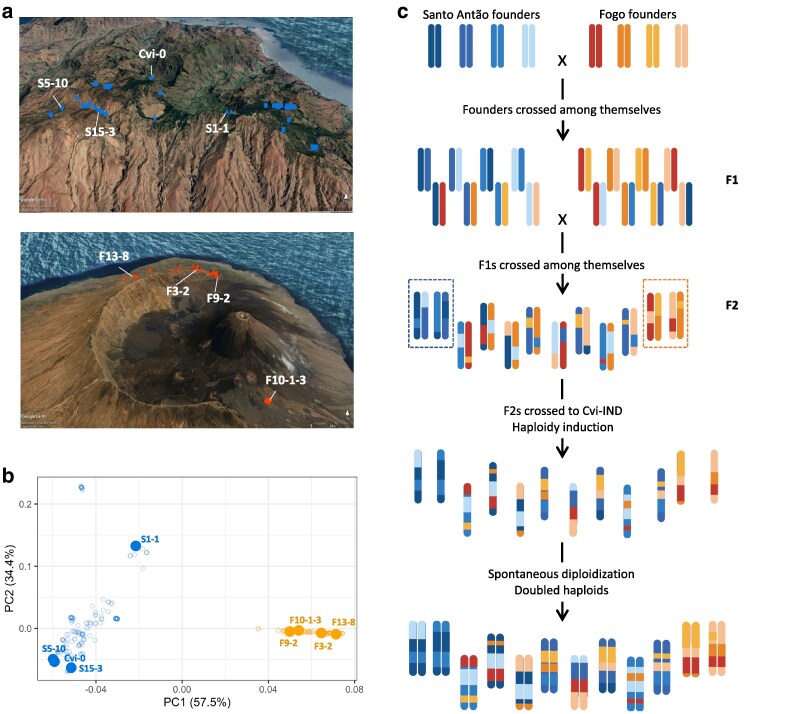
Construction of a Cape Verdean doubled haploid intercross population. a) Geographical location of the natural populations with the 8 founders labeled; top panel: Santo Antão, bottom: Fogo. b) PCA of the natural populations of Santo Antão (blue) and Fogo (orange). Each dot represents one accession, with larger dots and labels representing the 8 founders. c) Illustration of the crossing scheme for the production of the intercross population. Chromosomal segments in blue represent Santo Antão ancestry and in orange represent Fogo ancestry. Families contained within the dotted square represent within-island families. Chromosomes with blue and orange segments represent individuals from inter-island crosses. The 8 founders were crossed in all possible combinations, resulting in F1s that were subsequently crossed among themselves. The resulting dihybrid F2s were then crossed to the Cvi-IND line to produce haploid lines, which, after spontaneous genome doubling and phenotypic selection, resulted in 258 potential doubled haploid lines.

*A. thaliana* is present on 2 islands in Cape Verde: Santo Antão and Fogo. As a first step in creating a Cape Verdean inter-island doubled haploid (DH) population, we selected 4 accessions from Santo Antão (Cvi-0, S1-1, S5-10 and S15-3) and 4 from Fogo (F3-2, F9-2, F10-1-3, and F13-8) to represent genetic variation across the 2 islands (Fulgione et al. 2022). These 8 accessions are hereafter referred to as “founders” (Fig. 1a and b). We followed a half-diallel crossing scheme between all 8 founders, ie in all possible combinations, any 2 founders were crossed in a single direction (maternal and paternal accessions chosen at random) (Fig. 1c, Supplementary Fig. 1). These crosses were conducted both within each island and between islands, resulting in 16 inter-island F1s and 6 intra-island F1s for each island. In order to shorten linkage segments and to maximize allelic variation in the panel, these 28 F1s were further crossed among themselves (Supplementary Table 1), selecting for crosses between F1s that did not share any parents. The final number of resulting dihybrid F2 populations, and consequently DH lines, was dependent on crosses’ success and number of viable seeds per cross. We also limited the representation of each family to produce the most balanced design possible. The resulting set is composed by 3 families (F2 populations) within Santo Antão, 2 within Fogo, and 37 inter-islands families (with founders from both islands). Here, “family” refers to a set of DH lines with 4 founders in common.

Across experiments, all seeds used in the production of the DH intercross population including founders, F1s, F2s, and the inducer line, as well as in the phenotyping experiments, were stratified for 7 d at 4 °C in the dark prior to sowing. Because of previously identified nutrient transport issues in some CVI accessions, all plants used for the construction of the DH intercross population were grown under standard greenhouse conditions and supplemented with iron (Tergemina et al. 2022).

### Cvi-IND creation

To create a doubled haploid (DH) intercross population, we first needed to produce an inducer line carrying CVI cytoplasm (Cvi-IND) to avoid non-native nucleo-cytoplasmic interaction effects, as described in Flood et al. (2020) and Ravi et al. (2014). Briefly, the original Col-0 inducer line was used to pollinate several emasculated Cvi-0 flowers, so that the resulting offspring carried a cytoplasmic CVI background. Seeds from this cross were grown and diploid filial generation 1 (F1)—where genome elimination had failed—were selected and selfed. F2 seeds were propagated and plants exhibiting the inducer phenotype were selected and further selfed. Haploid inducer lines are characterized by relatively slow growth, semi-sterility, and wrinkled/lettuce-like rosette leaves (as described in Wijnker et al. 2014).

### Creation of the doubled haploid lines

To obviate the need to conduct several generations of selfing during the production of homozygous immortal populations, we used doubled haploid-inducing technology to produce homozygous lines (Ravi and Chan 2010; Seymour et al. 2012; Ravi et al. 2014).

For the crosses between the dihybrid F2 individuals generated (see above) and the Cvi-0 inducer line, the inducer line was used as the female progenitor. Because the haploid inducer line is only weakly male-fertile (Ravi and Chan 2010), no emasculation was needed. Fully developed flowers from each of the F2 individuals were crossed to a single Cvi-0 inducer line (Cvi-IND), which were then isolated to prevent undesired crosses and contaminations. Seeds resulting from these crosses can be diploid, aneuploid, haploid, or even self-pollinated inducers. Early in the development, haploid *Arabidopsis thaliana* present smaller and narrower rosette leaves compared to diploids, and, after bolting, haploid flowers are very small and sterile (Wijnker et al. 2014). We selected plants displaying phenotypes previously found to be associated with haploidy from the total set of progeny. By spontaneous genome doubling, some haploid plants will produce diploid siliques, which are fully developed and contain normal diploid seeds. Genotypes were validated from selected lines to determine whether they represented doubled haploid individuals.

### Sequencing and filtering

We genotyped 258 DH lines by whole-genome sequencing using an Illumina Hi-Seq3000 machine with 150 bp pair-end reads and an average coverage of 4×. Genomic DNA was extracted using BioSprint kits (Qiagen), following the manufacturer's instructions. Libraries were prepared using a TPase-based DNA assay.

The GATK pipeline (McKenna et al. 2010; Poplin et al. 2017) was used to genotype SNPs in the entire DH population based on high-coverage, high-confidence variant calls on the parents (Fulgione et al. 2022). The full pipeline is available at https://github.com/HancockLab/CVI/tree/master/genotype_calling/SNP_and_Indel_calling_GATK4. In short, reads were mapped to the reference genome TAIR10, using the Burrows-Wheeler Aligner (*bwa mem*) (Li, 2013), to an overall average mapping read of 98.46% (± 0.6%). Duplicates were marked with picard (*picard.jar MarkDuplicates*) (Picard Toolkit 2019), and variants called and genotyped with GATK using the flags *HaplotypeCaller*, *GenomicsDBImport*, and *GenotypeGVCFs*, in this order (Poplin et al. 2017). We retained only biallelic sites and used the following filters: *QualByDepth* < 2, Quality <30, *StrandOddsRatio* > 3, *Fisher Strand* > 60, *RMSMappingQuality* < 40, *MQRankSum* < −12.5 and *ReadPosRankSum* < −8. This resulted in a total of 18,287 SNPs.

### Imputation and curation

To ensure that all DH lines selected were in fact the result of spontaneous genome doubling and were therefore homozygous lines, we also identified SNPs *de novo* and excluded individuals with more than 1% heterozygosity across the genome (Supplementary Fig. 2a). This step reduced the final DH set from 258 to 214 lines.

To identify DH lines resulting from crossing or labeling mistakes, as well as possible uncontrolled outcrossing events, we imputed parental haplotypes in the DH lines using the option “*reconstruction*” in the MAGIC software (http://mtweb.cs.ucl.ac.uk/mus/www/19genomes/magic.html) (Kover et al. 2009). This software infers breakpoints from low-coverage sequence data, imputes missing data in the offspring based on the parental genotypes, and reconstructs genomic mosaics from known parental haplotypes. We used the log error probability of the imputation step to exclude individuals with genomic segments assigned to the incorrect founders, ie if a segment in the DH was assigned to a founder not used in the construction of that family, that DH line was removed. Five lines were identified as problematic, producing a final clean set containing 209 DH lines.

### Diversity and population structure in the DH population

We quantified diversity and assessed genetic structure in the natural population and in the DH population. Principal component analysis (PCA) was computed in PLINK (*−pca*) (Purcell et al. 2007). Nucleotide diversity was calculated per window, in both the DH population and the parents separately, using vcftools with the flag *–window-pi 1000* (Danecek et al. 2011).

### Phenotyping

#### Bolting time

We grew the final DH set (209 individuals) and the 8 founder lines in a growth chamber simulating several aspects of the Cape Verdean environment (hourly temperature and humidity, daylength, precipitation, and light intensity) as in Fulgione et al. (2022). DH lines were grown in 4 replicates while the founders were grown in 36 replicates in a randomized block design. A plant was considered to be bolting when the main stem reached 1 cm, and the number of days from sowing until bolting was recorded as the bolting time. Throughout the paper, bolting time is used as a proxy for flowering time.

#### Rosette area and photosystem II efficiency (ΦPSII)

We measured rosette size and photosystem II efficiency (ΦPSII) in the final doubled haploid CVI intercross population (4 replicates each), as well as 10 replicates of each of the 8 founders of this intercross panel. Plants were grown in a climate-controlled growth room with a high-throughput phenotyping system, the Phenovator, under conditions set to match a representative CVI site as closely as possible given technological constraints: 12/12 h day/night, irradiance 150 µmol m^−2^ s^−1^, 20/14 °C day/night temperature, with standard feeding solution Hyponex, with EDDHA-chelated iron at 150 µM. The Phenovator is a high-throughput phenotyping system in which 1,440 plants can be grown and imaged. The imaging system is based around a moving camera system that can image all plants multiple times per day, including at night. Pulse-amplitude modulated chlorophyll fluorescence imaging was used to calculate ΦPSII and projected leaf area (referred here as rosette size) (Flood et al. 2016; van Rooijen et al. 2017).

Plants were grown for 3 wk, in a complete randomized block design, on rockwool blocks, and imaged 5 times per day. The resulting images were processed on a custom-made analysis software (TTI Phenotyping Data Analysis Software, Wageningen University), masking the background and retrieving only pixels identified as plants.

### Trait mapping

To infer the genetic architecture of the traits of interest, we ran a Bayesian sparse linear mixed model (BSLMM) implemented in GEMMA (Zhou and Stephens 2012; Zhou et al. 2013). Markov chain Monte Carlo (MCMC) was run with 10,000,000 sampling steps and 2,500,000 burn-in iterations. Median, minimum, and maximum for the percentage of variation explained (PVE) and the number of variants with sparse effects (n.gamma) were calculated across 10 runs per trait.

We conducted trait mapping using 3 different approaches: a SNP-based approach, using GEMMA (Zhou and Stephens 2012), and 2 haplotype-based approaches: (i) using the software developed for the *Arabidopsis* MAGIC population (http://mtweb.cs.ucl.ac.uk/mus/www/19genomes/magic.html) (Kover et al. 2009) and (ii) using R/qtl2 (Broman et al. 2019) to deal with the complex crossing design. The approaches complement each other, by balancing the high resolution and precision of SNP-based mapping with the power and ability to capture linkage and untyped variants offered by haplotype-based approaches.

For the trait bolting time, we used days (from sowing) until bolting as the phenotype, while for rosette size and ΦPSII we mapped each measured timepoint, with 5 timepoints per day over 3 wk (*N* = 89).

SNP-based mapping was conducted in GEMMA using the option *-lmm 4*, after computing a kinship matrix (*-gk 1*) with all DH lines (Zhou and Stephens 2012). When necessary, we used the flag *-c* to add covariates to the model, ie functional variants genotypes. Prior to that, and to counterbalance the effect low-coverage sequencing might have on GWAS, we imputed all missing data in the DH using high-coverage, high-confidence calls on the parents (data from Fulgione et al. 2022). For this, we used the software Beagle v5.5 with default parameters (Browning et al. 2018).

For one of the haplotype-based approaches, we used a software designed specifically to infer breakpoints from low-coverage sequence data and to impute the genomes of the MAGIC lines based on founder genotype, and to then perform association mapping on the imputed genomes (Kover et al. 2009). The first step (imputation) is described above and mapping was computed using the option “*genome_scan’* with the flag *-h* to perform haplotype analysis. Since this software does not take covariates, when necessary, we have instead mapped the residuals after correcting the different traits for the covariate of interest.

Moreover, we also mapped the traits of interest using R/qtl2 (Broman et al. 2019). We used BEAGLE-imputed genotypes for the DH individuals and the calls from parents from (Fulgione et al. 2022) as input for the mapping function scan1() from the R package *qtl2*. Mapping was done on genotypic probabilities computed using the function calc_genoprob() and corrected with a kinship matrix, calculated with the function calc_kinship(). When covariates were considered, genotypes of the target SNP were added per DH line. Significance levels were assessed using the function scan1perm() over 1,000 permutations.

All Manhattan plots of the results were drawn using R and show the -log_10_(*P*-value) per SNP or haplotype across the genome for GEMMA and MAGIC results, and LOD score for R/qtl2.

### Statistical analysis

All statistical analyses were conducted in R. All phenotypes scored were analyzed using a linear mixed model approach, using the restricted maximum likelihood procedure with the *lme4* package (Bates et al. 2015 ) to obtain a single value per genotype while correcting for position in the chamber. These values were used as the phenotype for mapping in each case.

In addition to considering all timepoints from the Phenovator for mapping, we also identified the timepoint at which trait variance in the DH population was maximized, across the 89 measurements obtained over the length of the experiment, using PCA (function PCA() from the package *FactoMineR* [Lê et al. 2008]). Based on this analysis, we identified day 14 (timepoint 14.65, specifically, 7 h after the lights were turned on in the growth chamber and corresponding to the afternoon) as the timepoint with highest contribution for both ΦPSII and rosette size.

We tested for transgressive segregation using a Dunnett's test (package *DescTools* [Signorell 2020]) on raw phenotypes to include replicates per genotype. Differences in parental contribution for each DH line were tested using Pearson's Chi-squared test, with simulated *P*-values based on 2,000 replicates. Combination of *P*-values from SNP- and MAGIC haplotype-based approaches was calculated using the Brown's method, implemented with the function empiricalBrownsMethod() from the package *EmpiricalBrownsMethod* (Poole 2017). Comparisons between different genotypes to assess alleles effects were done using the Wilcoxon test or a linear model. Functions and packages used are included in https://github.com/celianeto/DH.

## Results

### Genetic variation among the DH lines

**Fig. 2.**
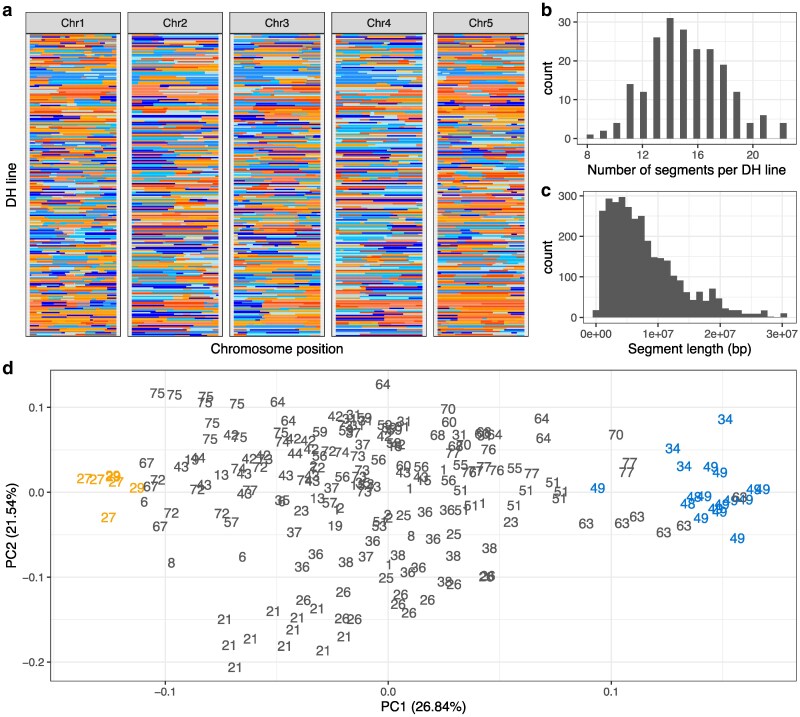
Genomic composition of the final doubled haploid intercross population. a) Genomic segments ancestry in the DHs. Each row represents 1 DH line (y-axis). Parental contribution is shown per genomic segment across chromosomes (x-axis). Colors represent the 8 founders (blue shades show contribution from the 4 Santo Antão accessions, orange shades from the 4 Fogo accessions). b) Distribution of the number of segments per DH line in the final population. c) Distribution of genomic segment length in base pairs (bp) in the final population. d) PCA with the final intercross population. Each point represents 1 DH line and the numbers correspond to the family number (same number, same set of founders). Blue marks families with all 4 founders from Santo Antão, orange marks families with all 4 founders from Fogo, gray inter-island crosses.

We produced a doubled haploid intercross population using founders from Santo Antão and Fogo, the 2 Cape Verdean islands (Fig. 1, Supplementary Fig. 1). The final DH intercross population was composed of 209 lines (Fig. 2, Supplementary Fig. 2, Supplementary Table 1), distributed across 42 families, with a median of 4 DHs per family (Supplementary Fig. 2b; min = 1, max = 13), resulting from 184 inter-island crosses, 7 within Fogo and 18 within Santo Antão.

As expected given the limited number of crossing events, short range linkage disequilibrium (LD) is stronger and decays more slowly in our constructed DH population compared to the natural populations (Supplementary Fig. 2d). Each DH genome has on average 15 genomic segments (Fig. 2b; min = 8, max = 22) with an average length of 7.8 Mb per segment (Fig. 2c; min = 146 Kb, max = 30 Mb, median = 6.5 Mb). Each DH line contains a roughly equal contribution from each of its 4 founders (Supplementary Fig. 2c; mean = 0.13, min = 0.09, max = 0.14), with no significant differences in contribution among founders (Pearson's chi-squared test, *X*^2^ = 0.010458, *P*-value = 1).

Consistent with the limited number of founders, diversity is reduced in the CVI intercross relative to the natural population. The DH population carries a total of 18,287 segregating SNPs, compared to the natural population samples from Cape Verde, composed of 335 individuals across the 2 islands, which together carry 73,406 segregating SNPs, most of which are at low frequency in the populations (Fulgione et al. 2022). Overall, the pairwise nucleotide diversity of the DH population is lower than the natural population (mean across 1 Kb windows = 2.4 × 10^−4^, min = 9.1 × 10^−8^, max = 3 × 10^−3^, mean across 1 Kb windows in founders = 3.9 × 10^−4^, min = 1.3 × 10^−4^, max = 8.9 × 10^−3^, Wilcoxon test, *W* = 246,497,406, *P*-value < 2.2 × 10^−16^).

The main reason for producing the intercross population was to overcome issues related to population structure. Consistent with this goal, we found population structure was greatly reduced in the final intercross population relative to the natural populations. In a PCA, DH individuals are widely distributed across the first 2 principal components (PCs) (Fig. 2d). However, as expected, individuals belonging to the same family clustered more closely and intra-island families clustered tightly together within islands and were clearly separated along the first principal component axis in the PCA. Compared to the PCA from the natural populations, the difference is striking, with a more continuous distribution across the axes, for the intercross population (Figs. 1b and 2d). Inter-island families (shown in gray in Fig. 2d) clustered between the 2 intra-island clusters, as expected based on their mixed ancestry between the islands. The homogenization of the structure exhibited in the PCA implies that the DH mapping population can complement and extend the variation available for mapping in the natural populations.

### Phenotypic variation

We measured flowering time in the DH population and the 8 founders in a growth chamber under CVI-simulated conditions, and rosette size and ΦPSII in a high-throughput phenomics installation, the Phenovator (Flood et al. 2016). Because data from the phenomics installation were collected across many timepoints, we first identified the timepoint contributing most substantially to the differentiation between lines using PCA (day 14, 7 h after dawn) and used this timepoint for downstream analyses (Supplementary Fig. 3). These traits are correlated (Pearson's *R* = 0.25, *P*-value = 4.3 × 10^−13^). Broad-sense heritability (based on repeatability across replicates) was 0.88 for flowering time and 0.32 for rosette size and 0.54 for ΦPSII (based on the single timepoint that contributes the most to trait variation).

**Fig. 3.**
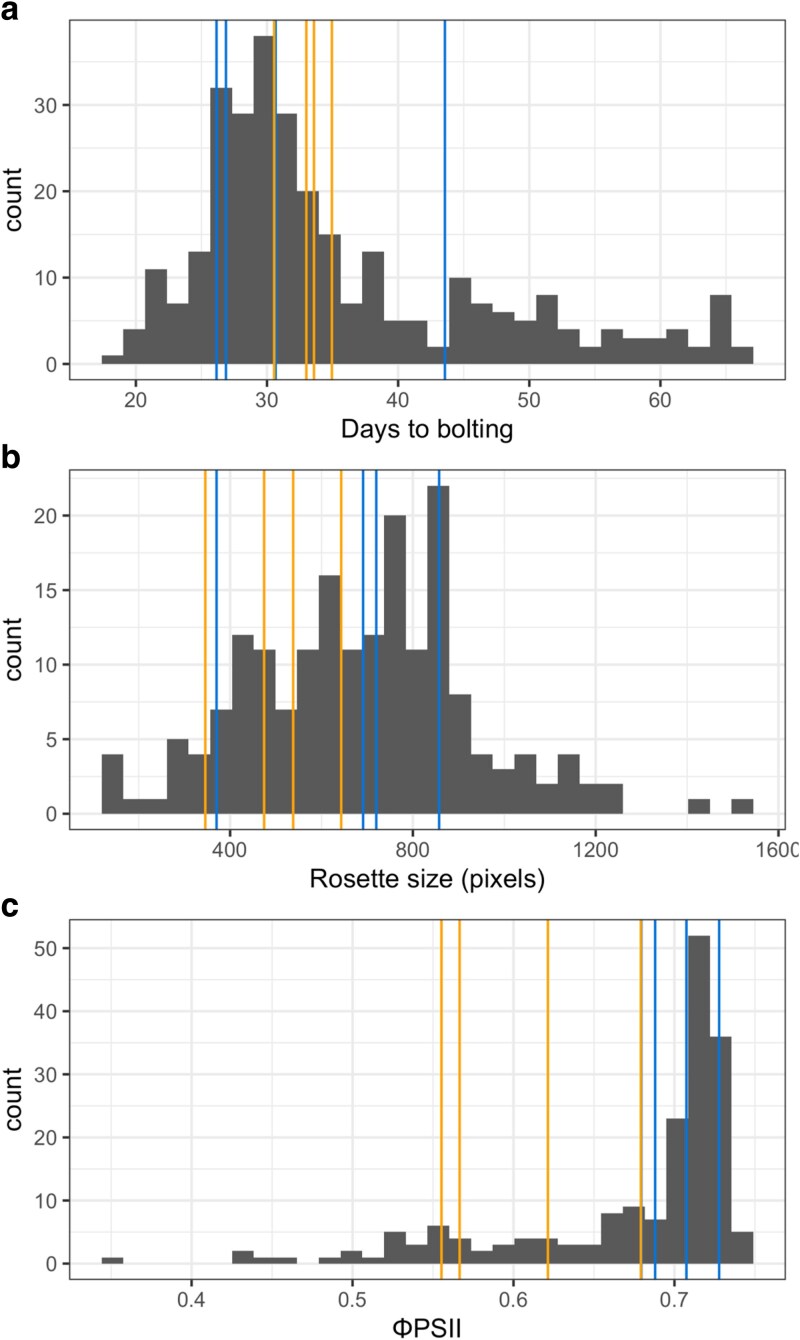
Phenotypic distribution in the intercross population for a) bolting time, b) rosette size, and c) ΦPSII. For b and c, the timepoint shown (14 d after sowing) contributes the most to trait variation. In each panel, DH individuals are shown in the histogram, and vertical lines show the median phenotypes for each founder (blue, Santo Antão founders; orange, Fogo).

On average, the DH population bolted later than the parents (35.4 vs 32.4 d), had larger rosettes (852 vs 580 pixels), and had higher ΦPSII (0.68 vs 0.65 Φ_PSII_) (Fig. 3, Table 1). However, the differences were not statistically significant (Wilcoxon tests, *P*-value _flowering_ = 0.861, *P*-value _rosette size_ = 0.194, *P*-value _ΦPSII_ = 0.138). The lack of significance was likely due to the increased trait variance in the DH population relative to the founders (Fig. 3, Table 1). Consistent with this, we observed transgressive segregation in the DH population for all traits (Dunnett's test, *P*-value _flowering_ = 5.6 × 10^−5^, *P*-value _rosette size_ = 0.0024, and *P*-value _ΦPSII_ = 0.0101). The higher trait values in the DH population and greater trait variance relative to the founders could be due to complementation of different derived alleles that arose independently in the 2 CVI islands.

**Table 1.** Summary of phenotypic variation in the DH intercross population and the founder lines.

Phenotypes	Founders range	DHs range	Founders mean ± SD	DHs mean ± SD	*P*-value Dunnett's transgressive segregation
Bolting (d)	[26; 44]	[18; 66]	32.4 ± 5.5	35.4 ± 11.3	5.6 × 10^−5^
Rosette size (pixels)	[345.7; 857.4]	[123.6; 1502.1]	580 ± 179	852 ± 249	0.0024
Φ_PSII_	[0.555; 0.728]	[0.352; 0.744]	0.65 ± 0.06	0.68 ± 0.07	0.0101

Mean, standard deviation (SD) and range (minimum and maximum) per trait are shown, as well as *P*-value for test on transgressive segregation.

### Trait mapping

We characterized the genetic architecture of the measured traits using a BSLMM implemented in GEMMA (Zhou and Stephens 2012; Zhou et al. 2013), estimated the percentage of variation explained by the genotyped markers (PVE) and inferred the number of loci contributing to the phenotype (Table 2, Supplementary Fig. 4). Bolting time showed the highest PVE and the greatest number of loci, implying a polygenic basis. Rosette size and ΦPSII showed relatively lower values compared to bolting but comparable between the two. Interestingly, ΦPSII showed a greater variation both for PVE and number of contributing loci across time (Supplementary Fig. 4), suggesting that photosynthetic efficiency may be dependent of the growth stage.

**Table 2.** Inferred genetic architecture of the traits mapped.

	PVE	Number of loci
Bolting	0.770 [0.747; 0.794]	17.08 [13.63; 22.34]
Rosette size	0.498 [0.211; 0.534]	4 [3;18]
ΦPSII	0.597 [0.217; 0.766]	4 [3; 10]

For each trait, percentage of variation explained (PVE) and estimated number of loci contributing to the phenotype are shown. These values are medians calculated across 10 MCMC runs in BSLMM. In brackets, minimum and maximum values are shown.

We mapped these 3 traits in the DH population using a SNP-based linear mixed model (Zhou and Stephens 2012) and an haplotype-based model, specifically designed for mapping in multiparent intercross MAGIC *Arabidopsis* populations (Kover et al. 2009). We further mapped the traits of interest using R/qtl2 (Broman et al. 2019) to deal with our complex crossing design. Overall, results were concordant across mapping approaches. The combination of these approaches takes advantage of the SNP-based high resolution and specific associations with traits, while the haplotype-based approach considers groups of linked variants, capturing broader patterns of inheritance and improving power to detect associations, especially when causal variants are untyped or weakly tagged.

**Fig. 4.**
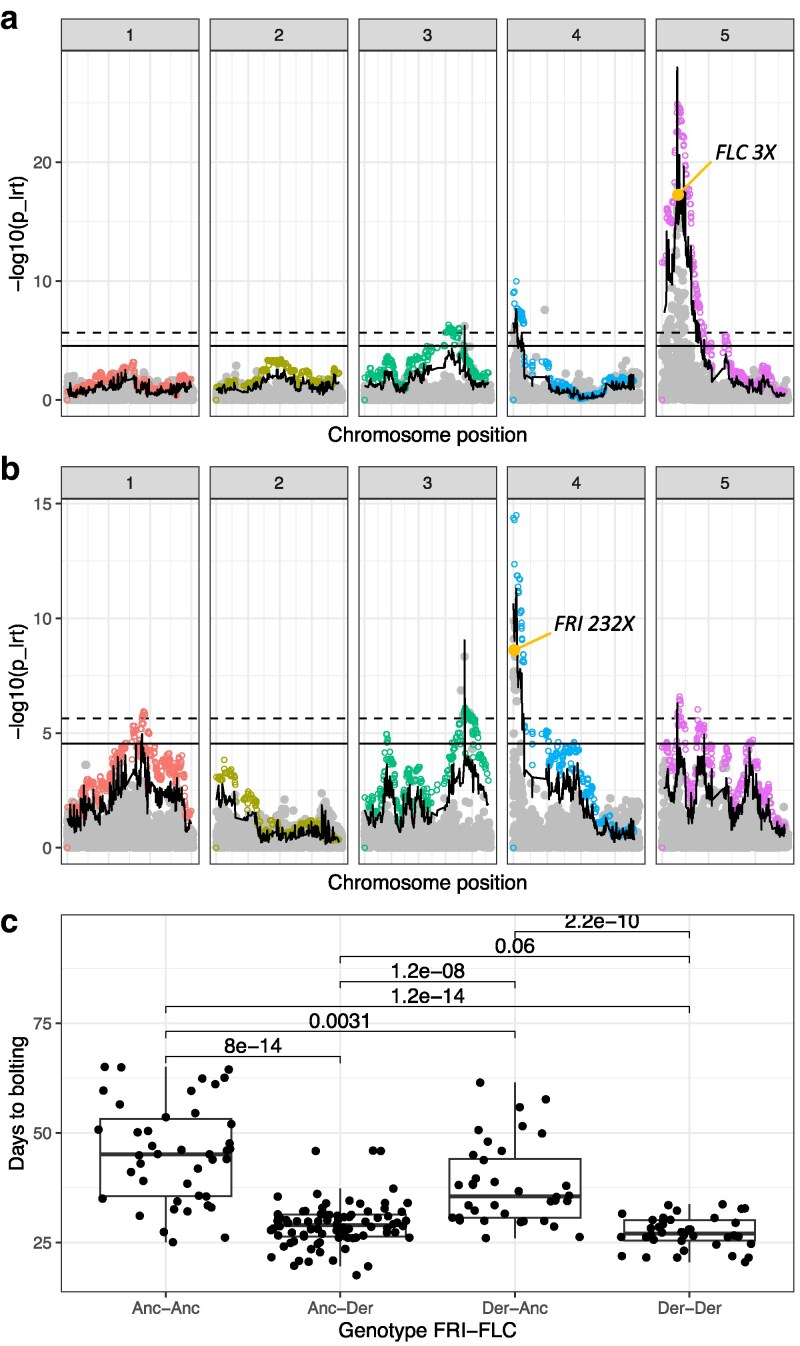
Flowering time mapping in the DH intercross population. Manhattan plots for (a) all variants segregating in the population and (b) the model corrected for the effect of *FLC* 3X. On both plots, the x-axis shows genomic positions across chromosomes and the y-axis the log_10_ transformed *P*-values for association (*p_lrt*). Filled gray dots represent SNPs used for the SNP-based approach, while open colorful dots represent haplotypes used in the MAGIC haplotype-based approach. The wavy black line represents the Brown's combined *P*-value across approaches, while the horizontal ones show the Bonferroni significance cut-off (full: SNP-based approach, dashed: haplotype-based approach). Highlighted in yellow the candidate variants. c) Effect of functionally validated variants in flowering time (*FRI K232X* and *FLC R3X*) in the doubled haploid intercross population. “Anc” represents the ancestral and “Der” the derived allele in Cape Verde. Only significant *P*-values are shown for Wilcoxon test.

For bolting time, across methods, we identified 3 Bonferroni-significant QTL which together explain 84.1% of the genetic contribution to the trait variance (Fig. 4, Supplementary Figs. 5 and 6 and 7). The most significant peak, on chromosome 5, co-localizes with *FLC*, a transcription factor that functions as a repressor of floral transition, by imposing a vernalization requirement, and contributes to temperature compensation of the circadian clock (Lee et al. 1994; Michaels and Amasino 1999; Salathia et al. 2006). In this gene, we identified a nonsense mutation at the third amino acid (*FLC R3X*, 3rd most significant SNP) that creates a premature stop codon. In the natural population of Fogo, *FLC 3X* is fixed, being therefore not detectable using GWAS, and it is also completely absent from Santo Antão. Previously, we identified this allele based on allelic segregation with flowering time in a recombinant population (F2) created from an inter-island cross (Fulgione et al. 2022).

When we corrected the models for the effect of *FLC 3X*, the most significant peak, on chromosome 4, co-localized with *FRI*, a major flowering time determinant conferring the vernalization requirement (Gazzani et al. 2003; Stinchcombe et al. 2004; Shindo et al. 2005). At this locus, we identified another nonsense mutation (*FRI K232X*, 7th most significant SNP), which also creates a premature stop codon, disrupting protein function. This mutation was initially identified in Cvi-0 (Gazzani et al. 2003) and was linked to its early flowering behavior. More recently, it was found to segregate in the natural population of Santo Antão, where it reduces flowering time in 34 d, but it is completely absent from Fogo (Fulgione et al. 2022). In the intercross population, DH individuals carrying the *FRI 232X* allele bolted on average 7 d earlier and individuals carrying the *FLC 3X* allele bolted on average 16 d earlier than individuals carrying both ancestral alleles (Fig. 4c). These results show that this new intercross population allows mapping in recombinants between the strongly structured island populations. The identification of both loci and their respective functional variants provide proof of concept that the DH mapping population can be used to identify causal loci. Here, using the DH population, we simultaneously identified signals for both the Santo Antão-specific *FRI 232X* allele and the Fogo-specific *FLC 3X* allele, which had not been possible so far in the natural populations due to lack of shared variation between the islands. Moreover, with this multiparent intercross population, alleles fixed (or at high frequencies) in each island can now be identified, whereas they would be missed in GWAS in the natural populations.

The third QTL, on chromosome 3, is not associated with any previously identified functional variant (Fig. 4a and b, and Supplementary Fig. 6). In the proximity (±10 Kbp) of the 2 Bonferroni-significant SNPs, we found potential candidates, including *PAP21* (AT3G52810) and *PAP22* (AT3G52820), 2 genes in the purple acid phosphatases family, which is linked to floral development and Fe and Mn homeostasis (H. Zhu et al. 2005); *BGAL2* (AT3G52840), a β-galactosidase involved in the modification of cell wall polysaccharides (Gantulga et al. 2008); *VBF2* (AT3G50080), an F-box protein involved in growth, development and auxin response (Schwager et al. 2007); and *PEN3* (AT3G50110), a phosphatase involved in response to osmotic and salt stress (Pribat et al. 2012). The most significant SNP in the QTL falls in the first intron of *PEN3* and segregates at 11% frequency in the DH population. While it is completely absent from Fogo, in the Santo Antão natural population, it segregates at only 3%. Its low frequency may explain why it was not identified in previous mapping efforts using the natural population (Fulgione et al. 2022; Neto and Hancock 2023). In this recombinant population, the derived allele delays bolting by 10 d (Supplementary Fig. 5).

A fourth QTL showed Bonferroni-significant association when using the MAGIC haplotype-based approach (Fig. 4). This locus on chromosome 5 underlies *PTM* (AT5G35210), a PHD transcription factor involved in chloroplast retrograde signaling, which perceives high light and mediates transcriptional repression of *FLC* through recruitment of *FVE*, a component of the histone deacetylase complex, thereby controlling flowering under high light (H. Wang et al. 2017).

To further dissect the polygenic basis of flowering time, we added both known functional variants separately to the models to improve power to identify other QTLs contributing to trait variation (Fig. 4b, Supplementary Figs. 6 and 7). When we corrected both models for *FLC 3X* (Fig. 4b), the MAGIC haplotype-based method identified 3 new QTLs: 1 in chromosome 1, underlying *BBX17* (AT1G49130), a floral transition repressor under long-day conditions that is regulated by heat shock, interacts with the flowering time gene *CO*, and negatively regulates flowering time (Robson et al. 2001; Griffiths et al. 2003); and 2 additional loci on chromosome 5, underlying *HUA2* (AT5G23150), which regulates *FLC* mRNA levels, as well as other MADS-box flowering time genes such as *FLM* and *MAF2* (Chen and Meyerowitz 1999; Doyle et al. 2005; Q. Wang et al. 2007), and *NADP-ME2* (AT5G11670), a malic enzyme whose over-expression mutants showed delayed flowering (Badia et al. 2015). When both *FLC* 3X and *FRI* 232X were added to the models (Supplementary Fig. 6), a single haplotype became significant, containing AT1G66480, a gene involved in chloroplast avoidance movement under intermediate and high light intensities (Kodama et al. 2010). The QTL on chromosome 1 showed marginal association also when using the R/qtl2 approach. Overall, our results present new candidates to underlying flowering time variation—and adaptation—in Cape Verde, and highlight the need to use complementary mapping approaches when uncovering the genetic bases of polygenic traits.

**Fig. 5.**
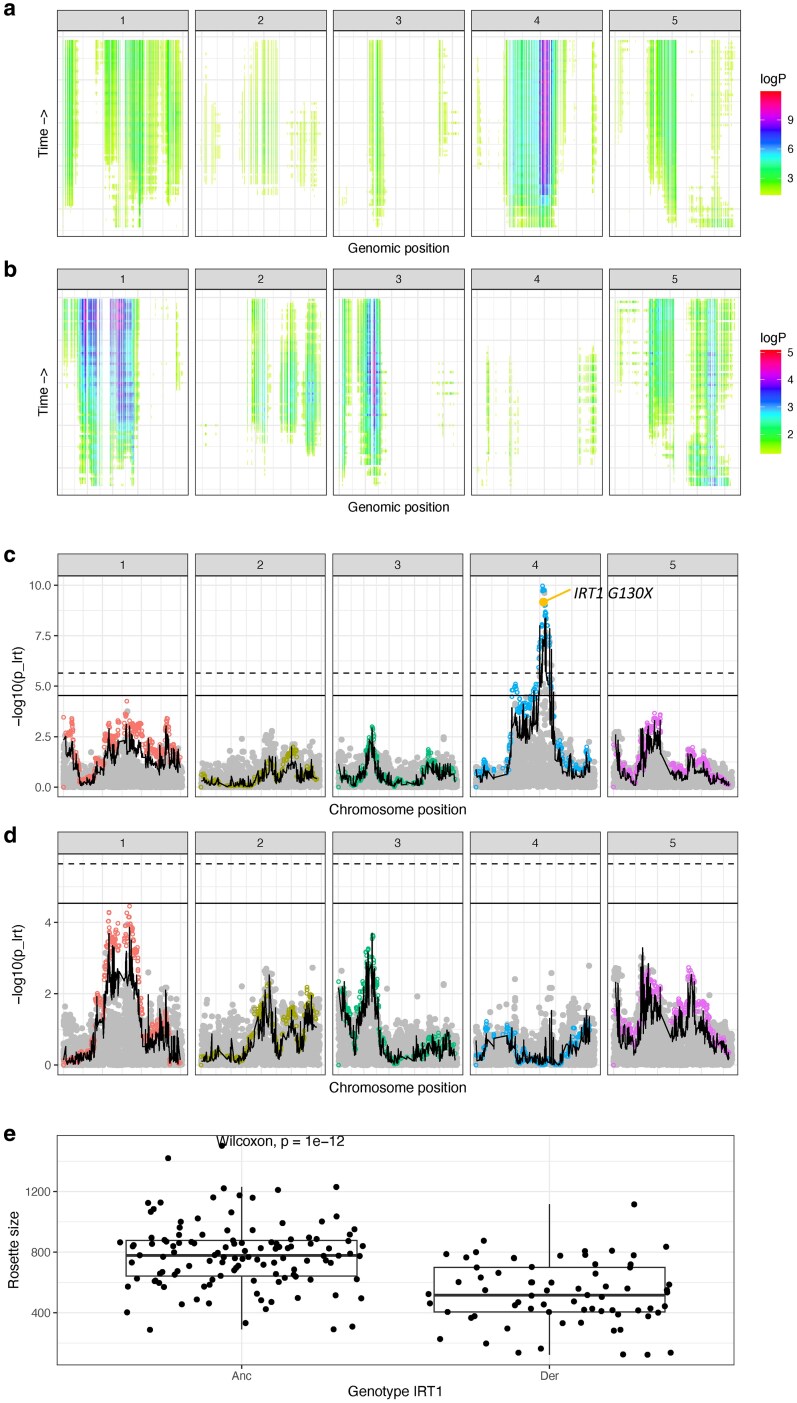
Rosette size mapping in the DH intercross population. a) and b) Heatmaps showing the MAGIC haplotype-based association. The x-axis shows genomic positions across chromosomes and the y-axis the different timepoints measured in the Phenovator platform (bottom represents the beginning of the experiment, top the end). For each haplotype at a different timepoint, color represents statistical association (only haplotypes with *P*-values < 0.05 are shown, *logP* refers to log_10_(*P*-values)). b) Heatmap shows mapping results when the model was corrected for the effect of *IRT1 G130X*, while a) shows the results for the full model. c) and d) Manhattan plots for a single timepoint (day 14). c) The full model, d) the results when *IRT1 G130X* was added as covariate. On both plots, the x-axis shows genomic positions across chromosomes and the y-axis the log_10_ transformed *P*-values for association (*p_lrt*). Filled gray dots represent SNPs used for the SNP-based approach, while open colorful dots represent haplotypes used in the haplotype-based approach. The wavy black line represents the Brown's combined *P*-value across approaches, while the horizontal ones show the Bonferroni significance cut-off (full: SNP-based approach, dashed: haplotype-based approach). Highlighted in yellow the candidate variants. e) Effect of the previously functionally validated variant *IRT1 G130X* (x-axis) on rosette size (y-axis) at day 14. “Anc” represents the ancestral and “Der” the derived allele in Cape Verde.

**Fig. 6.**
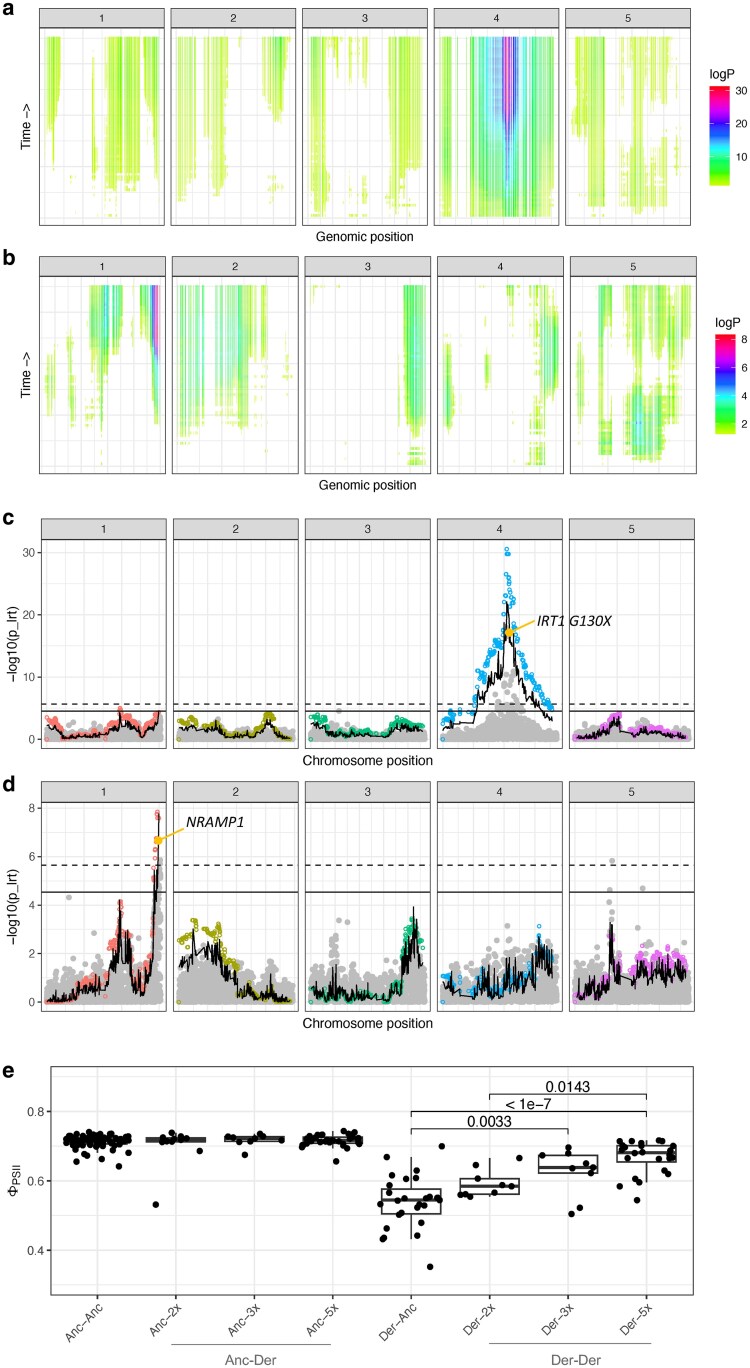
Photosynthetic efficiency (ΦPSII) mapping in the DH intercross population. a) and b) Heatmaps showing the MAGIC haplotype-based association. The x-axis shows genomic positions across chromosomes and the y-axis the different timepoints measured in the Phenovator platform (bottom represents the beginning of the experiment, top the end). For each haplotype at a different timepoint, color represents statistical association (only haplotypes with *P*-values < 0.05 are shown, *logP* refers to log_10_(*P*-values)). b) Heatmap shows mapping results when the model was corrected for the effect of *IRT1 G130X*, while a) shows the results for the full model. c) and d) Manhattan plots for a single timepoint (day 14). c) Manhattan plot shows the full model, d) the results when *IRT1 G130X* was added as covariate. On both plots, the x-axis shows genomic positions across chromosomes and the y-axis the log_10_ transformed *P*-values for association (*p_lrt*). Filled gray dots represent SNPs used for the SNP-based approach, while open colorful dots represent haplotypes used in the haplotype-based approach. The wavy black line represents the Brown's combined *P*-value across approaches, while the horizontal ones show the Bonferroni significance cut-off (full: SNP-based approach, dashed: haplotype-based approach). Highlighted in yellow the candidate variants. e) Epistatic interactions and the effect of *IRT1* and *NRAMP1* on ΦPSII (y-axis). X-axis shows combination of *IRT1* alleles and *NRAMP1* copy number (ancestral equals 1 copy) in this order. Throughout the figure, “Anc” represents the ancestral and “Der” the derived allele in Cape Verde. *P*-values refer to the linear model.

For mapping both rosette size and ΦPSII, we leveraged the time series phenotypic data, by mapping all timepoints to gain more power to identify associated loci (Figs. 5 and 6, Supplementary Figs. 8 to 11). Interestingly, we found that for both rosette size and ΦPSII, genetic architecture changed across time (Figs. 5 and 6, Supplementary Figs. 4 and 8 and 10). For rosette size, for example, some regions only showed association near the beginning of the experiment, while others showed signals only later. This suggests that different genes and variants play different roles over the course of plant development. This is further reflected by the changes in variation explained across time: rosette size showed an average PVE (percentage of variation explained, calculated with BSLMM implemented in GEMMA) of 49% with a standard deviation of 8%, while PVE for ΦPSII was on average 60% with a standard deviation of 18% (Table 2, Supplementary Fig. 4).

For rosette size, we identified a major peak of association on chromosome 4, consistent across time, both with the SNP- and the haplotype-based approaches (Fig. 5, Supplementary Figs. 8 and 9). This peak co-localizes with *IRT1*, a major gene in the iron transport pathway, for which we found a nonsense mutation (*IRT1 G130X*, 4th most significant SNP). Previously, we also found this truncating mutation associated with segregation for rosette size in a F2 population between a Fogo individual (also one of the DH founders) and the reference line Col-0 (Tergemina et al. 2022). This mutation was further validated with crosses and CRISPR mutant lines in the CVI background and linked to the small size observed in Fogo individuals (Tergemina et al. 2022). The mutation is fixed in Fogo, making it impossible to be identified in the natural population using GWAS, and completely absent from Santo Antão (Tergemina et al. 2022). Here, we showed that this can be done when making use of the multiparent intercross population, and were able to recapitulate the effect of the *IRT1 G130X* alleles, with the derived allele reducing rosette size (Fig. 5e).

When we corrected our mapping models for *IRT1 G130X*, we did not identify any other genome-wide significant candidate region (Fig. 5b and d, Supplementary Figs. 8b and 9b). However, other 2 regions showed a weaker association, consistent across time and methods, suggesting rosette size to be a less polygenic trait. These include a very broad region on chromosome 1, including the centromere, and another on chromosome 3, overlapping with *CAD4* (AT3G19450). CADs (cinnamyl alcohol dehydrogenases) act as the primary genes involved in lignin biosynthesis in *Arabidopsis thaliana*. A CAD double mutant showed a 40% reduction in lignin content in the main stem (Sibout et al. 2005), while a triple mutant (including a CCR mutant, another gene involved in lignin biosynthesis) presented dwarfism, characterized by smaller and fewer rosette leaves and shorter floral stem (Thévenin et al. 2011). These studies suggest *CAD4* could be a good candidate to underlying rosette size variation in Cape Verde.

Next, we mapped ΦPSII and, once again, identified a single peak of association on chromosome 4, consistent across time and methods, also co-localizing with *IRT G130X* (7th most significant SNP, Fig. 6, Supplementary Figs. 10 and 11). Previously, in a cross between a Fogo individual and Col-0, this variant was associated with size and leaf color, result of the low Fe levels in the leaves (Tergemina et al. 2022). Since Fe is required for core photosynthetic processes, it is not surprising to see its effect on ΦPSII, with the nonsense mutation causing a reduction in efficiency (Fig. 6e).

To further characterize the genetic architecture of ΦPSII and identify other genes contributing to its variation, we mapped the trait again, this time including *IRT1 G130X* as a covariate (Fig. 6b and d, Supplementary Figs. 10b and 11b). For timepoints 2 wk after sowing, we found a region at the bottom of chromosome 1 showing association, for both the SNP- and the haplotype-based approaches. At this locus, we identified as candidate *NRAMP1*, which encodes a metal transporter crucial for growth in Mn-deficient conditions that cooperates with *IRT1* for Fe transport (Curie et al. 2000; Cailliatte et al. 2010; Castaings et al. 2016). Since both nutrients are essential for photosynthesis, we considered this as the best candidate in the region. Moreover, *NRAMP1* had previously also been identified in association with the chlorotic phenotype observed in an F2 population between an individual from Fogo and Col-0 (Tergemina et al. 2022). In the natural population of Fogo, copy number variation of this gene was strongly correlated with greenness, rosette area, Fe in leaves, and relative chlorophyll content. This suggested that in Fogo the effect of *IRT1* disruption is partially rescued by the tandem duplications in *NRAMP1* (Tergemina et al. 2022). Here, making use of the variation between islands and its recombination, we can assess the effect of *NRAMP1* copy number variation in a CVI *IRT1* functional background (Fig. 6e). In the *IRT1 G130* background, we found no effect of *NRAMP1* copy number variation on ΦPSII (LM, *P*-value = 0.611). *IRT1 130X* on the other hand caused a decrease in phenotype of 0.20 (LM, *P*-value <2 × 10^−16^), which can be rescued by *NRAMP1* copies, with each extra copy increasing the phenotype by 0.03 units (LM, *P*-value = 1.12 × 10^−14^). This observation of a correlation between photosynthetic activity and copy number only in the disrupted *IRT1* background is consistent with our previous finding that the copy number variation at *NRAMP1* only has a detectable effect in the absence of *IRT1* function (Tergemina et al. 2022).

When the models were corrected for the large effect of *IRT1 G130X*, in addition to *NRAMP1*, other regions showed association with the trait (Fig. 6b and d, Supplementary Fig. 10b). Two of these are worth noting: the region in the middle of chromosome 1, which co-locates with the region found for rosette size, and the region at the bottom of chromosome 3, which co-locates with the third QTL found for flowering time. These results may suggest a deeper and more complex relationship across traits, and the effect pleiotropy may play in adaptation.

Overall, our results reveal the importance of complementary mapping approaches for uncovering the genetic bases of complex traits. Additional power for mapping may be acquired via time series phenotyping, informing us about the trait architecture changes across time. Furthermore, our results show the polygenic basis of different adaptive traits in the harsh Cape Verdean environment, despite low genetic diversity caused by the severe bottleneck associated with long-range colonization.

## Discussion

After colonization of the harsh Cape Verdean environment, the founder population adapted to the stronger selective pressures imposed by the shorter growing season and the challenging soil composition. In the inter-island DH population we produced, we mapped the genetic bases of 3 classes of potentially adaptive traits: rosette size and ΦPSII across developmental time and time to flowering, and we uncovered loci that contribute to the quantitative variation in these traits.

For flowering time, we identified large effect variants in the major flowering time regulators *FLC* and *FRI*, as well as new candidate loci. *FLC 3X* is Fogo-specific and fixed in the natural population, and therefore could not have been identified via GWAS in the natural population. *FRI 232X* was recently identified in the natural population of Santo Antão using GWAS (Fulgione et al. 2022) but had failed detection in previous studies using biparental recombinant populations between Cvi-0 and European lineages (e.g., Cvi-0×L*er*-0) because both parents carried a non-functional (weak) allele (Alonso-Blanco, El-Assal, et al. 1998; Shindo et al. 2005; Schmalenbach et al. 2014). Here, we showed that this new multiparent inter-island mapping population enables identification of both variants simultaneously. Moreover, by combining approaches, both at the SNP and haplotype level, we were able to identify additional candidate genes linked to flowering time variation in Cape Verde. Our findings highlight the importance of complementary approaches in detection the genetic basis of polygenic complex traits.

By mapping ΦPSII and rosette size in this intercross population, we identified *IRT1 G130X*, a variant fixed in the natural population of Fogo (Tergemina et al. 2022). We also identified another locus linked to ΦPSII and co-localizing with *NRAMP1*, making it a strong candidate to underlie variation in photosynthetic efficiency since both nutrients are essential for photosynthesis. This finding is supported by work in the natural population of Fogo, where copy number variation at *NRAMP1* was found to increase Mn levels in leaves and to rescue the effect of *IRT1* disruption on Fe transport (Tergemina et al. 2022). Using the intercross population presented here, we were able to identify *IRT1 G130X* and *NRAMP1* loci together, and to assess the epistatic effects of both loci in a randomized CVI genetic background. Further, by leveraging the time series phenotyping data we collected, we showed that the genetic architecture of ΦPSII changes over time, with the effect of *IRT1* present across the entire duration of the experiment, while the effect of *NRAMP1* is more noticeable only at later timepoints.

A major advantage of this intercross population is the break-down of population structure relative to the natural population (Churchill et al. 2004; Huang et al. 2011; King et al. 2012; Srivastava et al. 2017; Scott et al. 2020). In this case, this allows for variants that in nature are private to islands or sub-populations within islands to be identified in mapping and to have their effects assessed in novel combinations. In CVI, the 2 island populations were founded from a single ancestral population and separated early. The populations evolved almost exclusively from new variation that arose after the founding and initial separation of islands, so that trait architecture then evolved independently on the 2 islands (Fulgione et al. 2022). Recombinant progeny between the 2 islands—as represented in the intercross population we present here—allows for the reconstruction of a hypothetical ancestral population, in which island-specific alleles and their respective ancestral states are combined, making it possible to understand the deeper history of traits of interest. Moreover, with these recombinants we are able to assess the effects of these different alleles and their epistatic interactions in a randomized CVI genetic background, sharing a similar ancestry, no longer confounded by very divergent genetic backgrounds. Ultimately, this recombinant mapping population can enable the identification of genomic regions involved in adaptive divergence, revealing how different selective pressures shaped each population. By bringing together ancestral variants that have been separated through colonization of new local environments or even eliminated from the population, we can begin to recapitulate the first moments of colonization.

Future extensions of this work could integrate *A. thaliana* lines originating at different levels of geographical and demographic separation to produce an even more comprehensive understanding of the genetic steps that led to adaptation. For instance, at a worldwide scale, additional mapping populations from across the distribution of *A. thaliana* could provide information about the genetic basis of adaptation across the species distribution. Moreover, other CVI intercross populations that include the closest outgroup population from Morocco could fill the gap between divergent and CVI-specific variation, similarly to what has been done with other European lineages but using a closer ancestral population.

The Cape Verde population of *A. thaliana* has served as a model for determining the genetic basis of complex traits and their evolution after colonization of a novel habitat. Here, we described a new panel of genetically diverse recombinant lines of *A. thaliana* that highlights the importance of employing complementary approaches when mapping adaptive variation. This doubled haploid intercross population provides an additional layer to the available set of resources. The contribution of the new intercross population is especially important for variants that are confounded with population structure in the natural population. Together, these resources will enable more comprehensive dissection of the genetic basis of complex traits and their evolution during adaptation to a challenging environment.

## Supplementary Material

iyaf227_Supplementary_Data

## Data Availability

Scripts used to conduct all analyses are publicly available at https://github.com/celianeto/DH. Genomic data for founder individuals was previously published and is deposited in the European Nucleotide Archive (ENA) under accession code PRJEB39079 and in NCBI SRA under the project number PRJNA802114. Data generated in this study, namely, the final VCF with all DH individuals, is deposited in the European Variation Archive (EVA) at EMBL-EBI under accession number PRJEB85918 (https://www.ebi.ac.uk/eva/?eva-study=PRJEB85918). Seeds for the CVI doubled haploid intercross population are available through NASC (NASC ID: N2112896, with lines from N2112679 to N2112895). Supplemental material available at GENETICS online.
