# Supplementary material for: Uncovering adaptation with a new Arabidopsis thaliana multiparent intercross population
Source: Genetics. 2026 Jan 13;232(2):iyaf227. doi: 10.1093/genetics/iyaf227 (PMC13181408; doi:10.1093/genetics/iyaf227)
Supplement: iyaf227_Supplementary_Data [file iyaf227_supplementary_data.zip › Figure_S10_GENETICS-2025-308465.pdf]

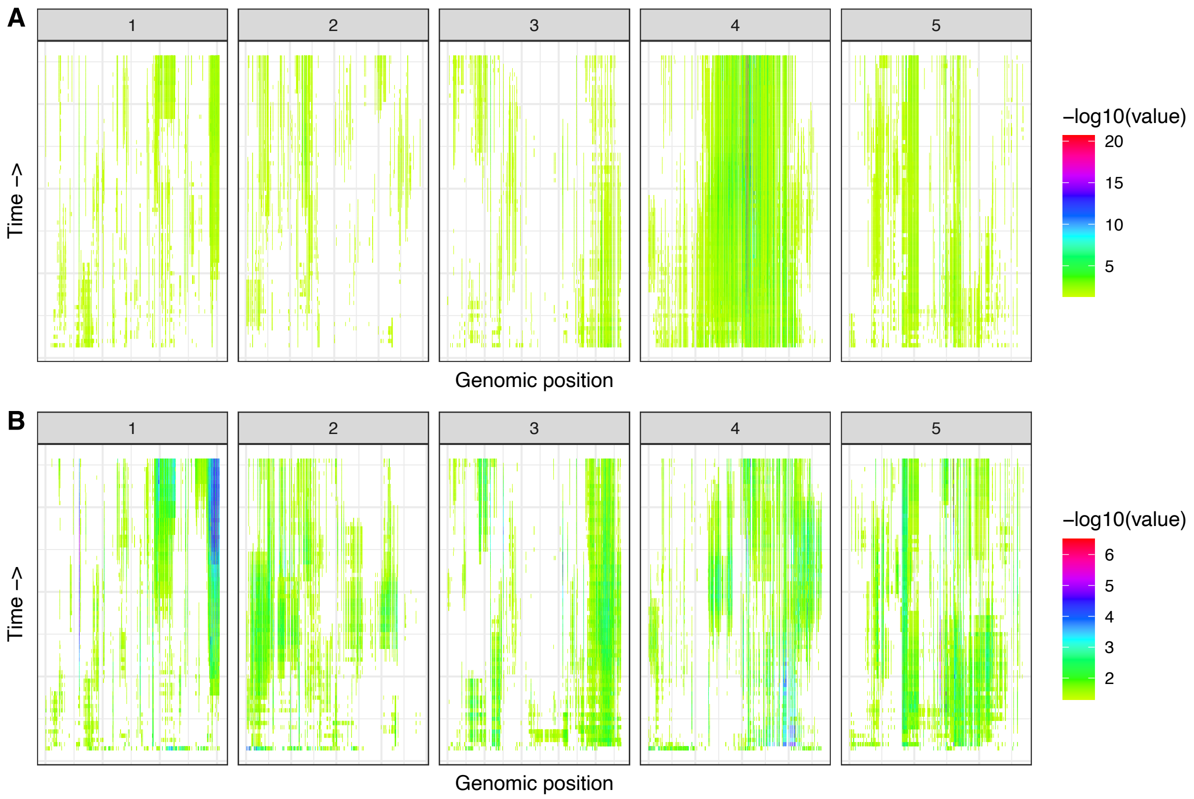

Figure S10. Photosynthetic efficiency mapping in the DH intercross population. Heatmaps showing SNP-based association. The x-axis shows genomic positions across chromosomes and the y-axis the different timepoints measured in the Phenovator platform (bottom represents the beginning of the experiment, top the end). For each SNP at a different timepoint, color represents statistical association (only SNPs with p-values < 0.05 are shown). A. shows mapping results for the full model, while B. shows the model corrected for the effect of *IRT1 G130X*.
