# Supplementary material for: Uncovering adaptation with a new Arabidopsis thaliana multiparent intercross population
Source: Genetics. 2026 Jan 13;232(2):iyaf227. doi: 10.1093/genetics/iyaf227 (PMC13181408; doi:10.1093/genetics/iyaf227)
Supplement: iyaf227_Supplementary_Data [file iyaf227_supplementary_data.zip › Figure_S1_GENETICS-2025-308465.pdf]

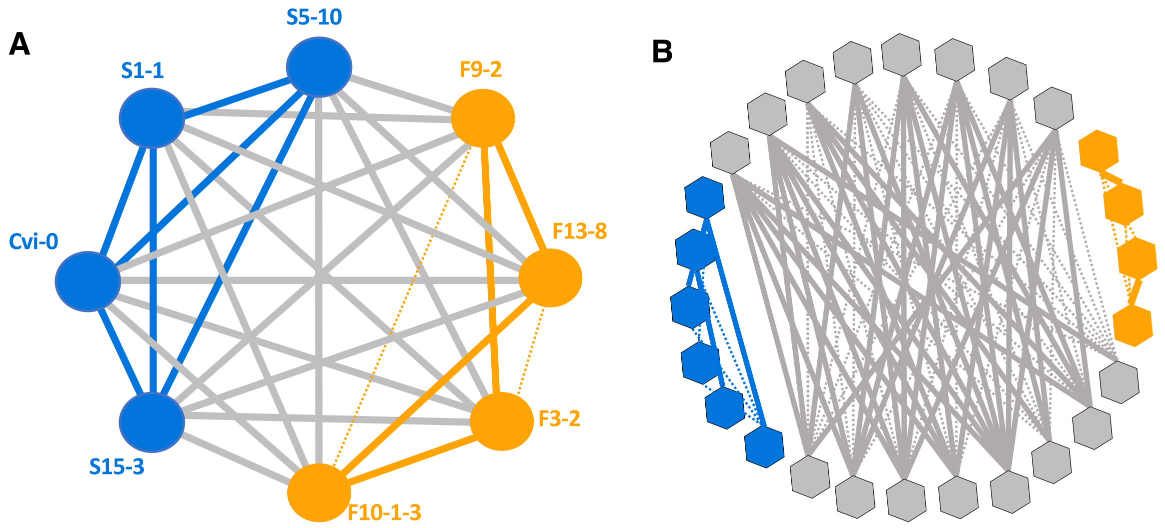

Figure S1. Construction of the multiparent intercross doubled haploid mapping population. A. All combinations of possible F1 crosses. B. Schematic of crosses to obtain F2 individuals, which would then be crossed to the haploidy inducer line (Cvi-IND). Throughout the figure, blue shows intra-Santo Antão crosses and families, orange intra-Fogo, grey inter-islands. Dashed lines mark crosses not represented in the final population.
