# Supplementary material for: Uncovering adaptation with a new Arabidopsis thaliana multiparent intercross population
Source: Genetics. 2026 Jan 13;232(2):iyaf227. doi: 10.1093/genetics/iyaf227 (PMC13181408; doi:10.1093/genetics/iyaf227)
Supplement: iyaf227_Supplementary_Data [file iyaf227_supplementary_data.zip › Figure_S2_GENETICS-2025-308465.pdf]

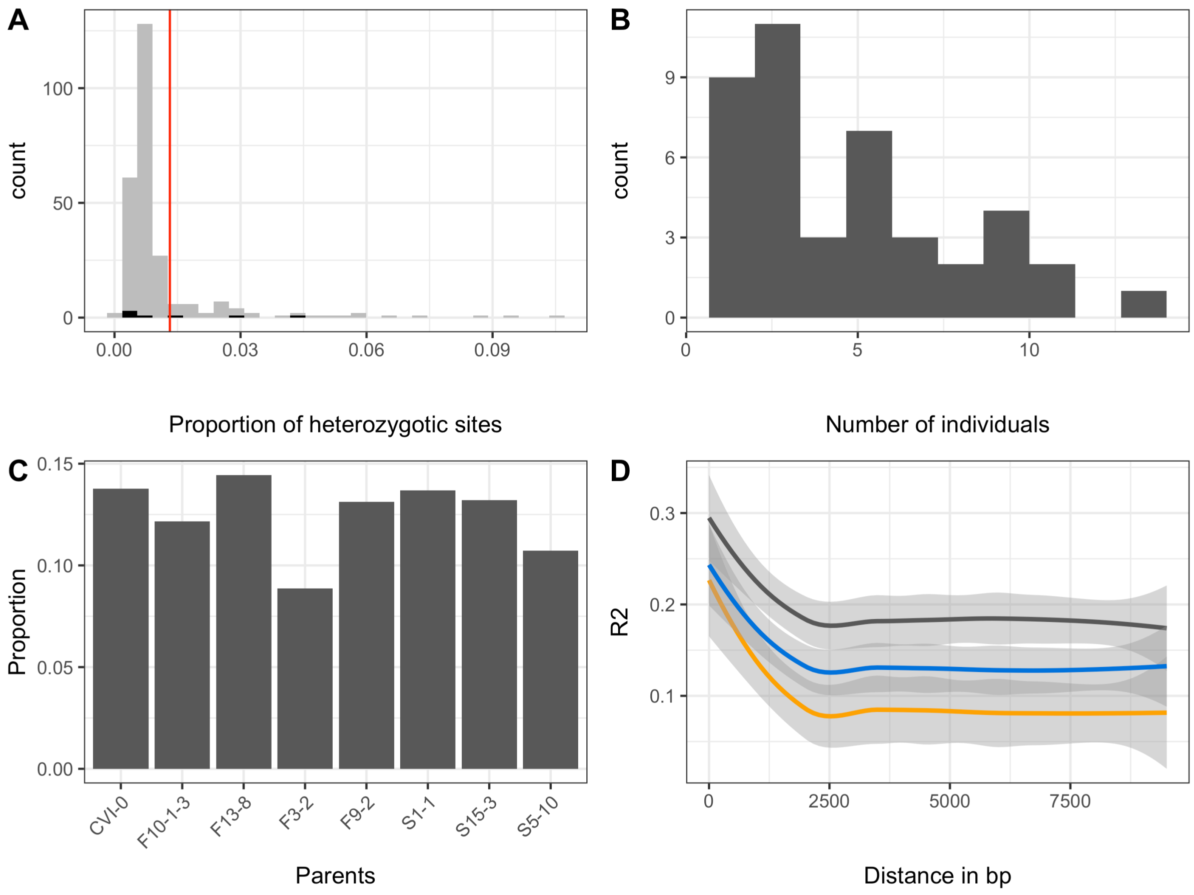

Figure S2. Statistics on the final doubled haploid population. A. Histogram of the proportion distribution of heterozygotic sites per DH line (in grey) and founder line (in black). The vertical red line marks 0.01 and the cut-off for a DH line to be considered in the final set. B. Histogram with the number of DH lines per family (x-axis). C. Proportion (y-axis) of each founder line (x-axis) contributing to the final DH population. E. Comparison of LD decay rates in 10 Kbp windows – R^2 between any two variants (y-axis) and their distance in bp (x-axis) – between the natural populations of Santo Antão (in blue) and Fogo (in orange), and the DH population (in grey). Loess smoothed lines are shown, with light grey areas representing standard error.
