# Supplementary material for: Uncovering adaptation with a new Arabidopsis thaliana multiparent intercross population
Source: Genetics. 2026 Jan 13;232(2):iyaf227. doi: 10.1093/genetics/iyaf227 (PMC13181408; doi:10.1093/genetics/iyaf227)
Supplement: iyaf227_Supplementary_Data [file iyaf227_supplementary_data.zip › Figure_S3_GENETICS-2025-308465.pdf]

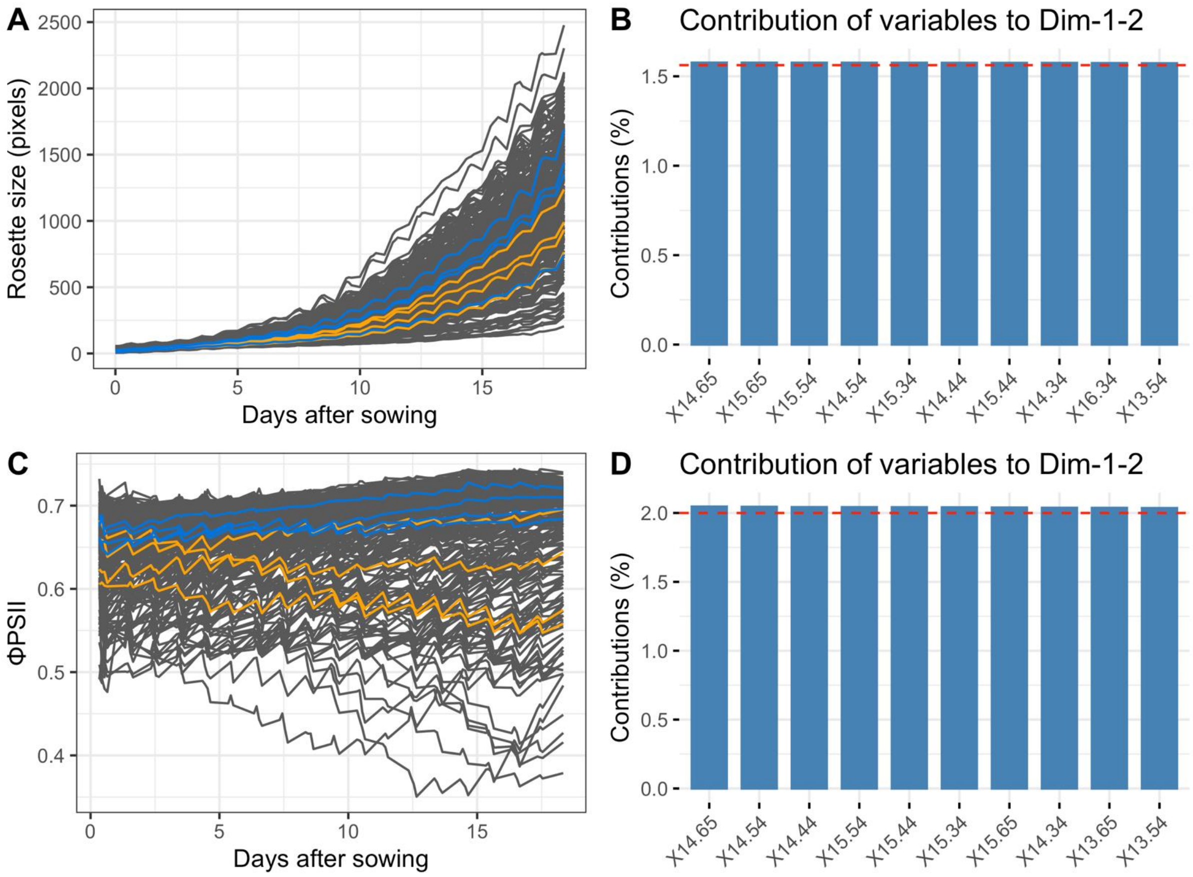

Figure S3. Rosette size and ΦPSII across time (A and C, respectively). Y-axes show values per line and x-axes time. Each grey line represents one DH line, blue lines represent the Santo Antão founders and orange the Fogo. B and D show contribution (y-axis) of the top 10 timepoints (x-axis) to variation in, respectively, rosette size and ΦPSII.
