# Supplementary material for: Uncovering adaptation with a new Arabidopsis thaliana multiparent intercross population
Source: Genetics. 2026 Jan 13;232(2):iyaf227. doi: 10.1093/genetics/iyaf227 (PMC13181408; doi:10.1093/genetics/iyaf227)
Supplement: iyaf227_Supplementary_Data [file iyaf227_supplementary_data.zip › Figure_S4_GENETICS-2025-308465.pdf]

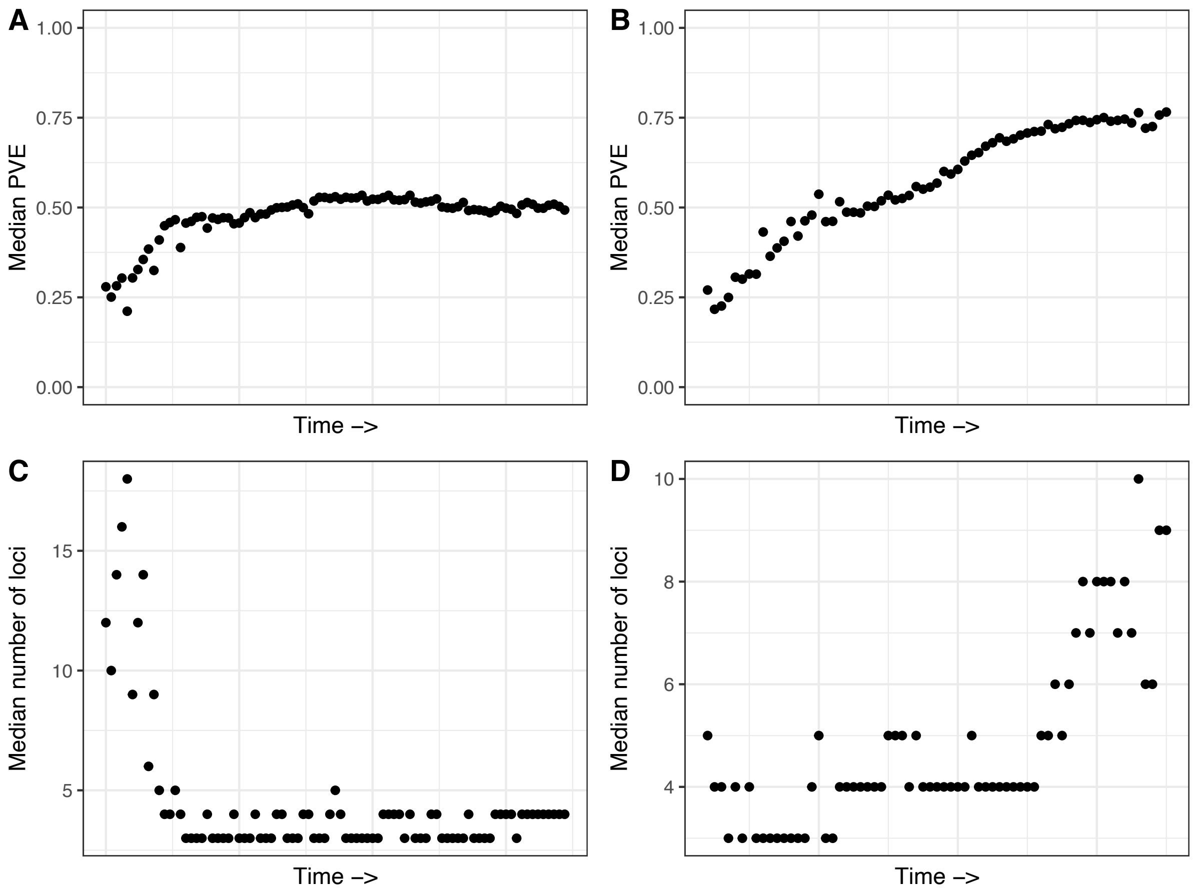

Figure S4. Inferred genetic architecture of rosette size (left, A and C) and ΦPSII (right, B and D) across time. Median percentage of variation explained (PVE, y-axis) across time (x-axis), for rosette size (A) and ΦPSII (B). Median number of contributing loci (y-axis) across time (x-axis) for rosette size (A) and ΦPSII (B). In each panel, each dot represents the median across 10 BSLMM runs.
