# Supplementary material for: Uncovering adaptation with a new Arabidopsis thaliana multiparent intercross population
Source: Genetics. 2026 Jan 13;232(2):iyaf227. doi: 10.1093/genetics/iyaf227 (PMC13181408; doi:10.1093/genetics/iyaf227)
Supplement: iyaf227_Supplementary_Data [file iyaf227_supplementary_data.zip › Figure_S5_GENETICS-2025-308465.pdf]

A

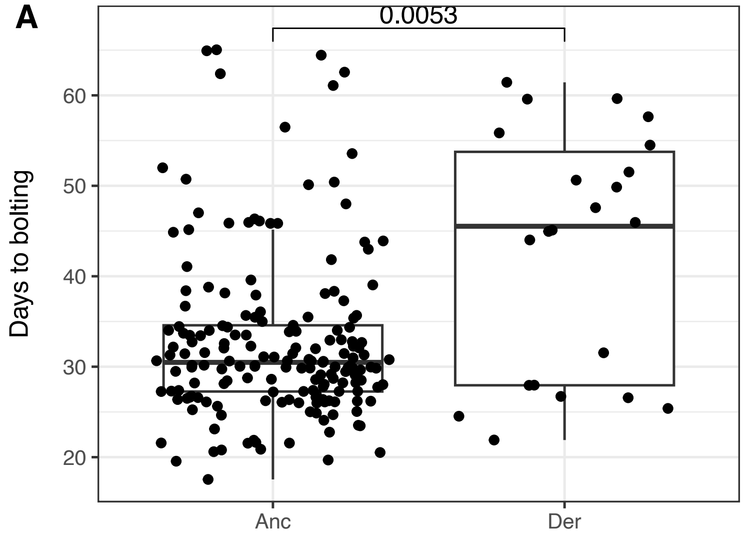

Figure S5. Effect of the most significant SNP identified by GWAS (chromosome 3) on bolting time mapping. Each dot represents one DH line. P-value from Wilcoxon test. X-axis shows the two alleles and the y-axis the phenotype. “Anc” refers to the ancestral allele and “Der” to the derived in Cape Verde.
