# Supplementary material for: Uncovering adaptation with a new Arabidopsis thaliana multiparent intercross population
Source: Genetics. 2026 Jan 13;232(2):iyaf227. doi: 10.1093/genetics/iyaf227 (PMC13181408; doi:10.1093/genetics/iyaf227)
Supplement: iyaf227_Supplementary_Data [file iyaf227_supplementary_data.zip › Figure_S6_GENETICS-2025-308465.pdf]

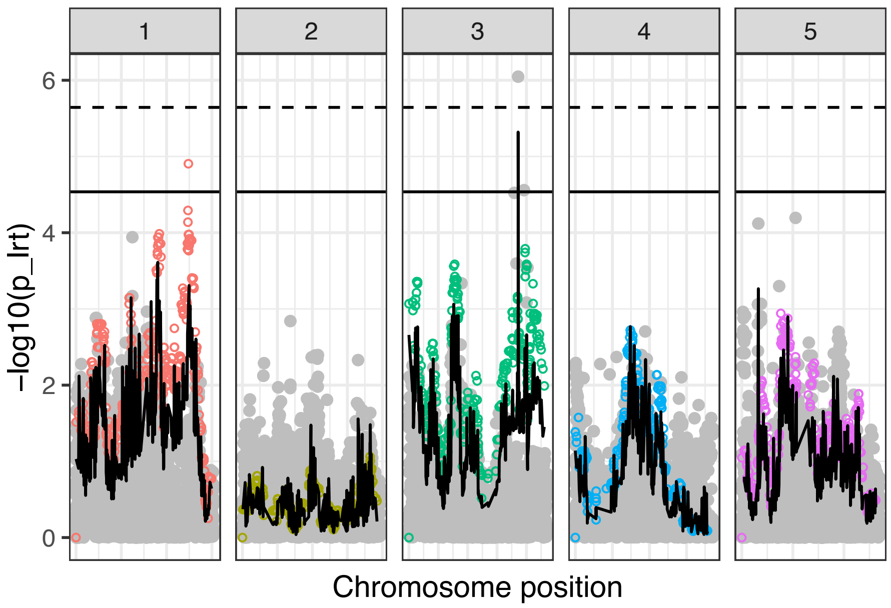

Figure S6. Flowering time mapping in the DH intercross population with ***FLC R3X*** **and** ***FRI K232X*** **as covariates.** Manhattan plot with results when the effect of both *FLC R3X* and *FRI K232X* are included as covariates in the models. X-axis shows genomic positions across chromosomes and the y-axis the $\log_{10}$ transformed p-values for association (*p_lrt*). Filled grey dots represent SNPs used for the SNP-based approach, while open colorful dots represent haplotypes used in the MAGIC haplotype-based approach. The wavy black line represents the Brown's combined p-value across approaches, while the horizontal ones show the Bonferroni significance cut-off (full: SNP-based approach, dashed: haplotype-based approach).
