# Supplementary material for: Uncovering adaptation with a new Arabidopsis thaliana multiparent intercross population
Source: Genetics. 2026 Jan 13;232(2):iyaf227. doi: 10.1093/genetics/iyaf227 (PMC13181408; doi:10.1093/genetics/iyaf227)
Supplement: iyaf227_Supplementary_Data [file iyaf227_supplementary_data.zip › Figure_S9_GENETICS-2025-308465.pdf]

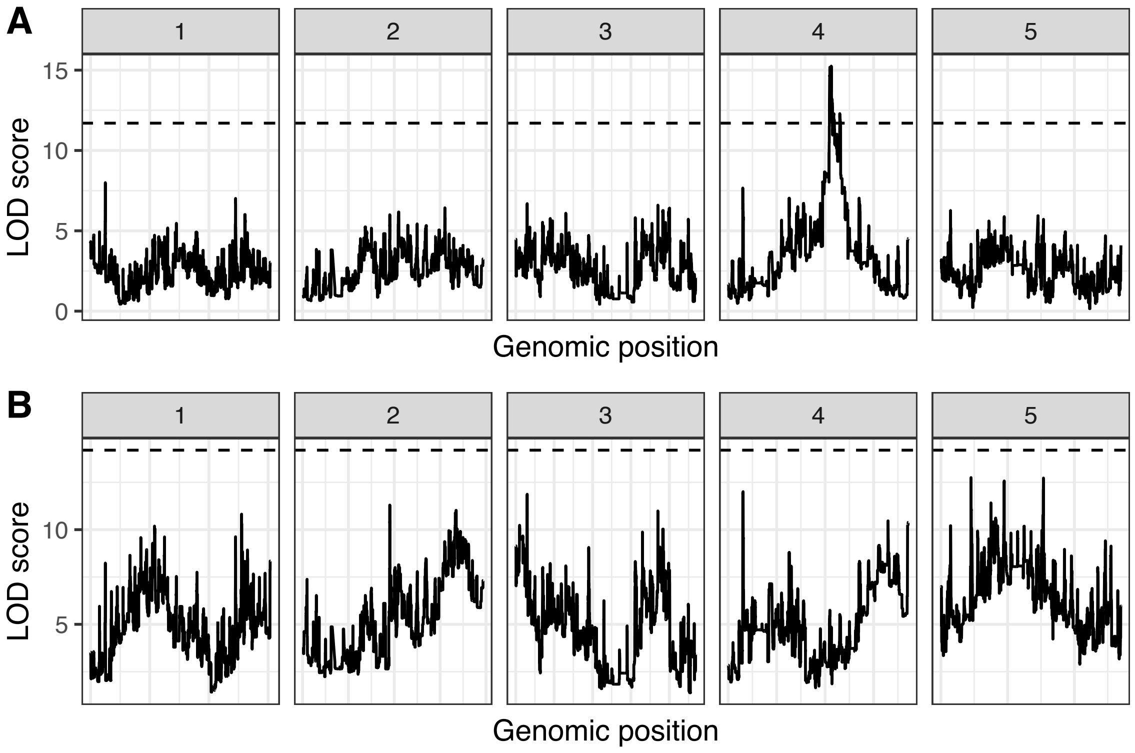

Figure S9. Rosette size mapping in the DH intercross population using the R/qtl2 approach. The two plots correspond to the model considering all variation (A), and the model corrected for *IRT1 G130X* (B). X-axis shows genomic positions across chromosomes and the y-axis the LOD score for association. The horizontal dashed line indicates significance, assessed with 1000 permutations.
